# Earthworm fauna of Catanduanes Island, Philippines, including descriptions of two new species of the genus *Pheretima* (Clitellata, Megascolecidae)

**DOI:** 10.3897/zookeys.1277.157678

**Published:** 2026-04-09

**Authors:** Yong Hong, Samuel W. James

**Affiliations:** 1 Department of Plant Medicine, College of Agriculture and Life Sciences, Jeonbuk National University, Jeonju 54896, Republic of Korea Department of Regenerative Organic Agriculture, Maharishi International University Fairfield United States of America; 2 Department of Regenerative Organic Agriculture, Maharishi International University, Fairfield, Iowa 52557, USA College of Agriculture and Life Sciences, Jeonbuk National University Jeonju Republic of Korea

**Keywords:** Biodiversity survey, sangirensis group, soil fauna, urceolata group

## Abstract

We describe new species of the genus *Pheretima* from Catanduanes Island, Philippines. *Pheretima lapulapui***sp. nov**. belongs to the *Pheretima sangirensis* group, with one pair of spermathecal pores in 7/8. *Pheretima portiae***sp. nov**. belongs to the *Pheretima urceolata* group, with one pair of spermathecal pores in 5/6. *Pheretima lapulapui***sp. nov**. has spermathecal pores 0.19–0.24 of the circumference apart and copulatory bursae openings 0.12–0.26 of the circumference apart. The intestinal origin in XVI, the vestigial typhlosole, the presence of a septum 8/9, and wedge-shaped pads surrounding the copulatory bursal openings separate *Pheretima lapulapui***sp. nov**. from other species. *Pheretima portiae***sp. nov**. is distinguished by spermathecal pores 0.16 of the circumference apart, copulatory bursae openings 0.24 of the circumference apart, unpigmented segmental equators, hearts in XI–XIII, and an intestinal origin in XV. Another taxon of the urceolata group, represented by a single individual, was briefly described but not named.

## Introduction

The Philippine’s Catanduanes Island lies east and north of the Bicol Peninsula, the southeastern extension of Luzon Island. The island is separated from Bicol’s Caramoan Peninsula by a 10 km strait. The general landscape of the island is hilly to mountainous, with elevations of less than 400 m that reach a maximum toward the central portion. Much of the island is covered with secondary forest, except for the coastal strip, where most of the human population lives. Our study focused on the center of the island in Summit Barangay (a barangay is the smallest political division in the Philippines system). Other indigenous earthworm species on Catanduanes Island include *Archipheretima gritzae* James, 2009, *Pleionogaster nautsae* James, 2006, and *Pl. viracensis* James, 2006, which we collected at the same time as those reported here.

The genus *Pheretima* Kinberg, 1867, is divided into two subgenera, *Pheretima* and *Parapheretima* Cognetti, 1912, based on the presence or absence of stalked secretory glands on the copulatory pouches. Most Philippine earthworms belong to the subgenus *P. Pheretima* without stalked glands according to recent faunal surveys (Hong and James 2011b), although some *Parapheretima* occur on the southern Philippine island of Mindanao (Aspe and James 2016). A preliminary report of the molecular phylogeny of some Philippine *Pheretima* indicated that the included sangirensis group species formed a clade, whereas all other provisional species groups in the analysis, including the urceolata group, were not monophyletic (James 2005; Aspe and James 2018).

Most of the high mountains in the Philippines, such as the northern Luzon Cordillera and several major ranges in Mindanao, host a high diversity of earthworm species. Our surveys of the earthworm fauna in these regions have typically yielded 5–10 species per location (James 2004a; Hong and James 2008a, 2008b, 2010, 2011a, 2011b, 2021; Aspe and James 2014, 2016, 2017), including *Pleionogaster* species and the pheretimoid genera *Pheretima* and *Pithemera* of the family Megascolecidae. We think that this diversity is typical of regions that are topographically diverse (e.g. Lee 1959) and that the mountainous regions in the Philippines are a rich natural reserve for the study of soil faunal diversity on relatively small geographic scales (Hong and James 2011a, 2011b). Although the topographic variability of Catanduanes Island is not as high as that of the aforementioned mountains, their earthworm diversity is comparable.

## Materials and methods

Specimens were collected through digging and hand-sorting soil, as well as by searching for leaf axils and other aboveground locations where organic matter accumulated. The specimens were killed in 50% ethanol and transferred to either 5% formaldehyde for long-term fixation or to 95% ethanol. The formaldehyde-fixed specimens were transferred to 80% ethanol after 48 h. Illustrations showing the anatomical views with important characteristics were prepared using a camera lucida. Morphological data were obtained via external examination and dorsal dissection under a microscope. Holotypes and paratypes were deposited in the National Museum of the Philippines’ Annelid Collection (**NMA**).

## Taxonomy

### Family Megascolecidae Rosa, 1891

**Genus *Pheretima* Kinberg, 1867**

#### Pheretima lapulapui sp. nov.

Taxon classification

Animalia

CrassiclitellataMegascolecidae

D5338299-4711-55F8-BD73-790EE56CF163

https://zoobank.org/A72D87D6-C44F-4C81-A3A5-31F7D1E6E8D4

Fig. 1, Table 1

##### Type material.

***Holotype***. Philippines • one clitellate Catanduanes Province, Catanduanes Island, near Barangay Summit, lowland forest, 13°46'N, 124°17'E; 275 m a.s.l., May 22, 2001; S.W. James leg. NMA 4813.

**Figure 1.**
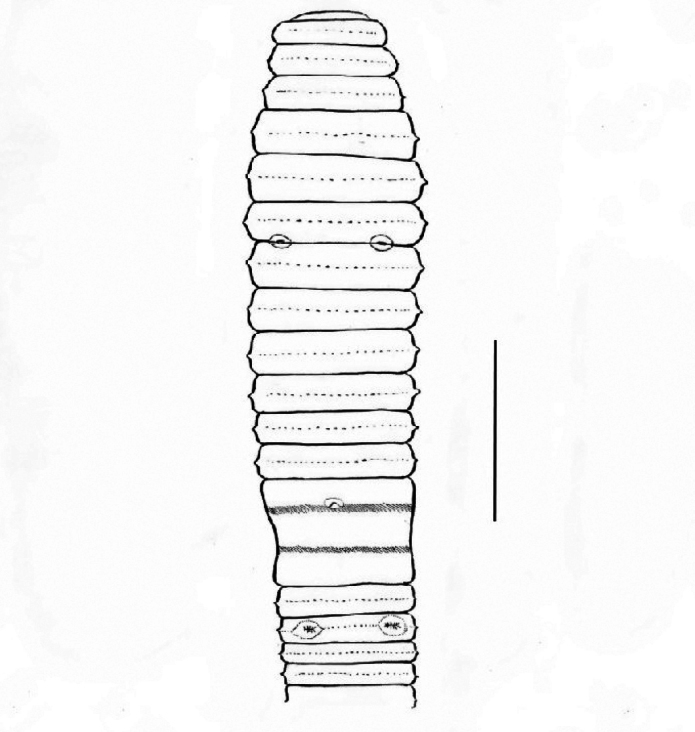
*Pheretima lapulapui* sp. nov.: ventral view. Scale bar: 10 mm.

**Table 1.** Comparison of species of the sangirensis group restricted to those with striped dorsal pigmentation, paired spermathecae in VII, intestinal origin in XVI, and (mostly) wedge-shaped pads surrounding the copulatory bursal openings. Several unpublished species are included with their location names. Setal counts are given as a pair of numbers separated by a comma, the first of the pair being the count in segment vii, and the second being the number in xx. Setal gaps are represented as D for dorsal gap, either present (+D) or absent (−D), and likewise V for ventral. Typhlosole presence is accompanied by the segment number of its origin if not near 27, the ordinary state. Septal presence and absence is indicated by + or −, the first of each pair being for septum 8/9. For bursal pads, those found on the floor of the bursa are represented by a number followed by F (e.g. 4F), and those attached to the roof of the bursa are represented by a number followed by R (e.g. 4R). Missing data are shown by a question mark (?). Data from Aspe and James (2017), Hong and James (2009), James (2004b), Hong and James (2025a, 2025b, 2026a), and Hong and James (unpublished data).

	*Pheretima virgata*	*Pheretima tigris*	*Pheretima bontocensis*	*Pheretima banahawensis*	*Pheretima taceae*	*Pheretima Kalahan* sp. 1	*Pheretima balerensis*	*Pheretima Aurora* sp. 1	*Pheretima lapulapui* sp. nov.	*Pheretima bulusanensis*	*Pheretima sorsogonensis*	*Pheretima blackbirdensis*
Setae vi, xxv	76, 80	38, 48–60	40, 62	46, 64	31, 36	42, 60	48, 64	34, 40	45–59, 72–74	43–47, 55–75	39–42, 45–50	52, 60
Setal gaps D, V	−D, −V	+D, −V	+D, +V	+D, +V	+D, −V	+D, −V	+D, −V	−D, −V	+D, +V	+D, +V	+D, +V	+D, +V
Male pore setae	4–5	0	4–6	4–7	1	11	2–6	3	13–15	10	4–6	14
Male pore spacing	0.09	0.14	0.10	0.09–0.13	0.13	0.20	0.12	0.12	0.17–0.22	0.19–0.23	0.13–0.14	0.17
Sp. pore spacing	0.11	0.13	0.09	0.09–0.13	0.12	0.11–0.15	0.08	0.10	0.19–0.24	0.24–0.27	0.14–0.17	0.20
Dorsal pores	12/13	12/13	12/13	4/5	5/6		11/12	11/12	12/13	12/13	12/13	11/12
Dorsal pigment	Brown stripes	Red-purple	Brown	Brown	Brown	Violet	Brown	Purple	Purple-black	Violet-brown	Light brown	Purple-brown
Equatorial pigment	None	None	None	None	None	None	None	None	None	None	None	None
Length, mm	290–360	230–280	68–83	130	80	94	85	105	132–200	116–138	56–68	85
Intestinal origin	xvi	xvi	xvi	xvi	xvi	xvi	xvi	xvi	xvi	xvi	xvi	xvi
Typhlosole	+	+	+	+	33	+	+	+	Vestigial	+	+	+
Esoph. lamellae	+	+	?	+	+	?	?	?	?	?	+	?
Intestinal vessels	32–42	56–58	28–30	30	?	30	36	32	34–38	?	30	42–44
Hearts	x–xiii	x–xiii	x–xiii	x–xiii	x–xiii	x–xiii	x–xiii	x–xiii	x–xiii	x–xiii	x–xiii	x–xiii
Septa 8/9/10	+, −	+, −	−, −	−, −	−, −	−, −	−, −	−, −	+, −	+, +	−, −	+, −
Bithecate vs mono	bi	bi	bi	bi	bi	bi	bi	bi	bi	bi	bi	bi
Thecal segm.	viii	viii	vii	vii	vii	vii	vii	Lvii, Rviii	vii	vii	vii	vii
Penes	+	−	+	+	+	+	+	+	−	+	+	+
Bursal pads	5F wedges 4R	5F wedges	4F wedges	5F, 1R	ring F	> 5F wedges	4F wedges	6F wedges	5F wedges	4F wedges	4F wedges	4 or 5F wedges

##### Additional material examined.

Philippines • two clitellates; same data as for holotype.

##### Type locality.

Philippines, Catanduanes Province, Catanduanes Island, near Barangay Summit, lowland forest 275 m a.s.l.

##### Diagnosis.

*Pheretima* species with one pair of spermathecal pores in 7/8 at 12^th^ setal line, deep in intersegmental furrow, spermathecal pores 0.19–0.24 of the circumference apart from each other; striped dorsal pigmentation, paired spermathecae in VII, intestinal origin in XVI, vestigial typhlosole, septum 8/9 present, and wedge-shaped pads surrounding the copulatory bursal openings.

##### Description.

Deep-purple to black dorsal pigment, absent from setal rings, producing a strong stripe pattern interrupted by dorsal setal gaps (larger worms) or not interrupted (smaller worms); ventral side with reddish stripes (larger worms) or unpigmented except striped head and tail segments (small worms). Dimensions 132–200 mm by 8.5–10 mm at segment X, 8–10.5 mm at XXX, 7–10 mm at clitellum, segments 83–118; body circular in cross-section. Setae numbering 45–59 at VII, 72–74 at XX, 13–15 between male pores, setal size equal between dorsal and ventral sides but spacing closer on ventrum, setal formula AA:AB:YZ:ZZ = 5:3:5:10.5 at XIII. Clitellum annular XIV–XVI; setae invisible externally. First dorsal pore: 12/13. One pair of spermathecal pores in 7/8 at 12^th^ setal line, deep in intersegmental furrow, 5–7.5 mm between spermathecal pores 0.19–0.24 circumference apart from each other. 0.3–0.6 mm openings of copulatory bursae paired in XVIII at 11^th^ setal line, 3.9–7 mm between openings, 0.17–0.22 circumference apart from each other. Genital markings were lacking.

Septa 5/6–7/8 muscular, 8/9 membranous, 9/10 absent, 10/11–12/13 thickly muscular, 13/14 thin gizzard in VIII, intestine begins in XVI; very small, paired lymph glands from XXVII along dorsal vessel; intestinal ceca simple originating in XXVII, extending anteriorly to XXIII, finger-shaped sac; typhlosole vestigial from XXVII, 34–38 intestinal blood vessels. Hearts in X–XIII; IX lateral; VIII to gizzard; VII connected to the ventral vessel.

Ovaries and funnels in XIII, one pair of spermathecae in VII with nephridia on ducts, spermathecae wrinkled ovate ampulla, barrel-shaped muscular duct shorter than ampulla, diverticulum pear-shaped iridescent chamber, and stalk shorter than ampulla. Male sex system holandric, testes, and funnels in paired ventral sacs at X–XI. Seminal vesicles, two pairs in XI–XII with apical dorsal lobe, prostates in XVIII with muscular ducts, curving around posterior side to enter upper posterior face of copulatory bursa without stalked glands; copulatory bursa openings surrounded by five wedges. The penis was bluntly conical with a male pore on the tip.

##### Differential diagnosis.

Table 1 includes the *P. sangirensis* group species with the following shared characteristics: striped dorsal pigmentation, paired spermathecae in VII, intestinal origin in XVI, and wedge-shaped pads surrounding the copulatory bursal openings. We included unpublished taxa from southern and central Luzon to facilitate comparisons in the table. Within this set of species *P. lapulapui* sp. nov. has the most setae between the male pores, generally more setae than the others, the widest male and spermathecal pore spacing, septum 8/9 present, and lacks a typhlosole.

##### Remarks.

*Pheretima lapulapui* sp. nov. shares morphological characteristics with the majority of the northern and central Luzon members of the species group, with paired spermathecae in VII, intestinal origin in XVI, and wedge-shaped pads surrounding copulatory bursal openings. *Pheretima lapulapui* sp. nov. is thus a slight geographical outlier because Catanduanes Island is closer to southern Luzon, adjacent to the Bicol Peninsula.

##### Etymology.

The species is named in honor of the Philippine national hero, Datu (chief) Lapu-Lapu, whose army defeated the Spanish at the Battle of Mactan on April 17, 1521.

#### Pheretima portiae sp. nov.

Taxon classification

Animalia

CrassiclitellataMegascolecidae

28254187-17E1-5A1C-BB1E-36FA3072BA61

https://zoobank.org/212F3D69-45AC-4395-AC9C-5EAD16399731

Fig. 2, Table 2

##### Type material.

***Holotype***. Philippines • one clitellate; Catanduanes province, Catanduanes Island, south of Barangay Summit on Virac-Viga Road, 13°47'N, 124°16'E, May 21, 2001, S.W. James leg. NMA 4814.

**Figure 2.**
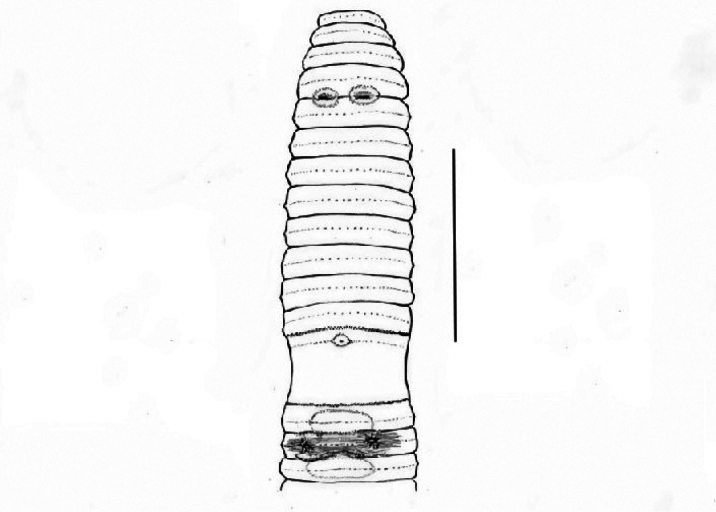
*Pheretima portiae* sp. nov.: ventral view. Scale bar: 5 mm.

**Table 2.** Comparison of species of the Philippine urceolata group. Setal counts are given as a pair of numbers separated by a comma, the first of the pair being the count in segment vii, and the second being the number in xx. Setal gaps are represented as D for dorsal gap, either present (+D) or absent (−D), and likewise V for ventral. Reproductive pore spacings are given as the estimated fraction of body circumference. Typhlosole presence accompanied by the segment number of its origin if not in XXVII. Septal presence and absence are indicated by + or −, the first of each pair being for septum 8/9. For bursal pads, those found on the floor of the bursa are represented by a number followed by F (e.g. 4F), and those attached to the roof of the bursa are represented by a number followed by R (e.g. 4R); those on the side are S. Missing data are shown by a question mark (?). Data from Aspe and James (2016, 2017), Hong and James (2008, 2009), and James (2004b).

	*Pheretima acia*	*Pheretima aquila*	*Pheretima baletei*	*Pheretima bukidnonensis*	*Pheretima davaoensis*	*Pheretima dinagatensis*	*Pheretima enormis*	*Pheretima gamay*	*Pheretima hamiguitanensis*	*Pheretima heaneyi*	*Pheretima lantapanensis*	*Pheretima libradoi*	*Pheretima kitangladensis*	*Pheretima monotheca*	*Pheretima urceolata*	*Pheretima nagaensis*	*Pheretima abiadai*	*Pheretima gorasi*	*Pheretima simsi*	*Pheretima portiae* sp. nov.	*Pheretima* sp. 1	*Pheretima bicolensis*	*Pheretima viracensis*	*Pheretima camarinensis*	*Pheretima batoensis*	*Pheretima buhiensis*	*Pheretima doriae*
Setae vi, xxv	26–35, 29–38	39–42, 44	24, 40	38, 52	44, 43–49	39, 43–48	45–46, 75–80	38–40, 36–45	38–42, 44–47	38, 43	30–39, 31–36	30, 59	44, 48	36, 32	20–25, 43	31–38, 41–48	32–42, 42–48	24–32, 25–41	31–33, 39–47	40–42, 41–44	31, 52	29–34, 49–55	37–38, 40–45	44–49, 48–52	36–43, 43–48	31–50, 66–70	35–40, 40–43
Setal gaps D, V	−D, −V	+D, −V	−D, −V	+D, −V	+D, −V	−D, −V	+D, −V	+D, −V	+D, −V	+D, −V	+D, −V	+D, +V	−D, −V	+D, −V	+D, −V	+D, +V	−D, +V	−D, −V	+D, +V	+D, −V	+D, −V	−D, +V	+D, −V	+D, −V	+D, +V	+D, −V	+D, +V
Male pore setae	3	7	6	10	10	12	7	1–5	2	6–8	8	0	8	3	2	6–9	6–9	0	6–8	4–6	6	2–3	7–10	6–9	6–8	0	5–8
Male pore spacing	0.24	0.21	0.24	0.17	0.27	0.24	0.14	0.13	0.12	0.19–0.23	0.23	0.10	0.20	0.1	0.11	0.25	0.19	0.10	0.26	0.24	0.14	0.14	0.20	0.19	0.15–0.18	0.07–0.12	0.32
Sp. Pore spacing	0.21	0.22	0.25	0.38	0.28	0.31	0.17	0.19	0.07	0.19–0.22	0.38	0.10	0.28	NA	0.16	0.29	0.25	0.06–0.07	~0.30	0.16	0.06	0.05	0.32–0.36	0.19	0.21–0.23	0.05	0.32
Dorsal pores	12/13	12/13	11/12	12/13	12/13	11/12	13/14	12/13	12/13	11/12	11/12	12/13	12/13	12/13	12/13	12/13	12/13	12/13	12/13	12/13	12/13	12/13	12/13	12/13	11/12	12/13	11/12
Dorsal pigment	Brown	Brown	Pale pink	Brown	Brown	Red-brown	Brown	Purple-brown	Brown	Brown	Brown	Purple-brown	Brown	Brown	Brown	Brown	Violet-red	Brown	Brown	Purple-brown	Purple-brown	Brown	Violet-brown	Light brown	Red-brown	Light brown	Red-brown
Equatorial pigment	+	None	+	Pale	+	None	None	+	None	None	+	None	None	+	None	None	None	+	+	None	Faint	+	+	None	Faint	+	None
Length mm	38–47	165	78	76	86–98	65	305–347	38–51	89–90	83–116	> 58	185	108	62	70–114	36–53	33–60	155–177	68–91	31–52	128	71–98	38–61	54–61	45–65	120–221	34–45
Intestinal origin	xv	xv	xvii	xviii	xvii	xiv or xv	xv	xvii	xv or xvii	xviii	xv	xv	xvi	xviii	xvi	xvi	xvi	xv or xvi	xv	xv	xvi	xvi	xvi	xv	xvi	xvi	xvi
Typhlosole	+	+	−	+	+	+	+	?	?	+	+	+	+	+	+	+	-	+	+	+	+	−	+	+	+	+	+
Esoph. Lamellae	+	+	+	+	+	+	+	+	+	+	+	+	+	+	+	+	?	+	?	?	?	?	?	?	?	?	?
Intestinal vessels	38	29	?	20	36	31–33	38	?	?	28	33	32	32	36	31	38	?	38	?	?	30–32	?	?	33–45	?	36	?
Hearts	x–xiii	x– xiii	x–xiii	xii, xiii	x–xiii	x–xiii	x–xiii	x–xiii	x–xiii	x–xiii	x–xiii	x–xiii	x–xiii	x–xiii	x–xiii	xi–xiii	xi–xiii	xi–xiii	x–xiii	xi–xiii	xi–xiii	xi–xiii	xi–xiii	x–xiii	xi–xiii	xi–xiii	xi–xiii
Septa 8/9/10	−, −	+, −	+, +	−, +	+,−	−, −	−, −	+, +	−, −	+, −	−, −	+, −	+, −	−, −	+, −	+, −	−, −	+, −	+, −	+, −	+, −	−, −	−, −	−, −	−, −	+, −	−,+
Bithecate vs mono	bi	bi	bi	bi	bi	bi	bi	bi	bi	bi	bi	bi	bi	mono	bi	bi	bi	bi	bi	bi	bi	bi	bi	bi	bi	bi	bi
Penes	+	−	+	−	+	+	+	−	−	+	−	−	−	+	+	+	+	+	+	+	+	+	+	−	+	+	+
Bursal pads	1F,1R	1F,2R	3F	0	2R	2R	2R	2R	1F	0	2R	1R	0	0	+R,+F	2S, 2F	2R	1F, 1S	2F	2F	3F	2F,1R	2S	2S	2F	1F, 2R	3S

##### Additional material examined.

Philippines • one clitellate, one aclitellate, same data as for holotype.

##### Type locality.

Philippines, Catanduanes province, Catanduanes Island, south of Barangay Summit on Virac-Viga Road, 13°47'N, 124°16'E.

##### Diagnosis.

One pair spermathecal pores in 5/6, spermathecal pores 0.16 of the circumference apart; copulatory bursae openings 0.24 of the circumference apart; unpigmented segmental equators; hearts XI–XIII, intestinal origin XV; copulatory bursae with two pads on floor and adjoining ovate glands protruding from anterior, posterior sides of copulatory bursae.

##### Description.

Medium purple-brown dorsal pigment absent on wide stripes at segmental equators. Dimensions 31–52 mm long, 2–2.5 mm wide at segment X, 3 mm at XXX, 3 mm at clitellum, segments 76–84; body circular in cross-section. Setae counts 40–42 at VII, 41–44 at XX, 4–6 between male pores, more widely spaced dorsally; setal formula AA:AB:YZ:ZZ = 2:2:2.5:4 at XIII. Clitellum annular XIV–XVI; setae invisible externally. First dorsal pore: 12/13. One pair of spermathecal pores in 5/6, 1 mm between spermathecal pores 0.16 of the circumference apart in 3^rd^ to 4^th^ setal lines, openings of copulatory bursa paired in XVIII, 1.5 mm between openings, 0.24 of the circumference apart in 6^th^ to 7^th^ setal lines. Genital markings lacking.

Septa 5/6–7/8 thick, 8/9 thin, 9/10 absent, 10/11–11/12 slightly thickened, 12/13 13/14 progressively thinner. Gizzard in VIII; intestine begins in XV; small paired lymph glands from XXVII along dorsal vessel; intestinal ceca simple originating in XXVII, and extending anteriorly to XXV; finger-shaped sac; typhlosole low, thick fold from XXVII. Hearts in XI–XIII; X absent; IX lateral; VIII to gizzard; VII lateral with small connections to anterior edge of gizzard.

Ovaries and funnels in XIII, spermathecae paired in VI with nephridia on ducts, spermathecae ovate ampulla; broad muscular duct shorter than ampulla, diverticulum chamber spherical with iridescence, sessile, or with a very short stalk at duct–ampulla junction. Male sexual system holandric, testes, and funnels in paired ventral sacs in X–XI. Seminal vesicles two pairs in XI–XII lacking dorsal lobes; prostates in XVII–XIX, 2 or 3 lobes wrapped in arc around copulatory bursae; straight muscular ducts, entering center of copulatory bursae without stalked glands; copulatory bursae openings flanked by anterior posterior oval pads connected to large glandular bodies clearly visible from body cavity, one each at anterior posterior edges of copulatory bursae; penis an oval transverse disk with a small ridge elevated laterally.

##### Differential diagnosis.

*Pheretima portiae* sp. nov. belongs to the urceolata group of Sims and Easton (1972), which comprises 26 *Pheretima* species, with spermathecal pores in 5/6. See James (2004b) regarding the history of synonymy among non-Philippine members for a review of the taxonomy of the group. Gates (1961) and Aspe and James (2017) identified some Philippine specimens as *P. urceolata*, which was previously known in Indonesia. We relied on the synthesis of *P. urceolata* morphological data from James (2004b) and Aspe and James (2017) in our comparisons of the new species. *Pheretima portiae* sp. nov. is the only member of the species group with unpigmented segmental equators, an intestinal origin in XV, and without hearts in X according to our morphology summary in Table 2.To further distinguish this species from others, the head setae count is approximately the same as the seta count in XXV rather than lower, and there are only two bursal pads on the floor. The two bursal pads have large gland masses that are visible within the body cavity as separate bulges protruding from the anterior and posterior ends of the bursae. The glands are broadly connected, not stalked, and appear to be bursae outgrowths. Luzon Island species of the urceolata group generally lack hearts in X, whereas those of Mindanao Island have hearts in X. An intestinal origin in XVI is the most common among Luzon species and varies from XV to XVIII in Mindanao.

##### Etymology.

The species is named after our chief coordinator and facilitator in the Philippines, Portia Nillos-Kleiven. She navigated institutional policies, procedures, and the people responsible for issuing permits for biodiversity surveys.

#### Pheretima

Taxon classification

Animalia

CrassiclitellataMegascolecidae

sp. 1

AA9EE8DC-62FF-5391-9ECC-394A7DB5179A

Fig. 3, Table 2

##### Material examined.

Philippines • one clitellate: Catanduanes province, Catanduanes Island, south of Barangay Summit on Virac-Viga Road, 13°47'N, 124°16'E, 21 May 2001, S.W. James leg. NMA 4815

**Figure 3.**
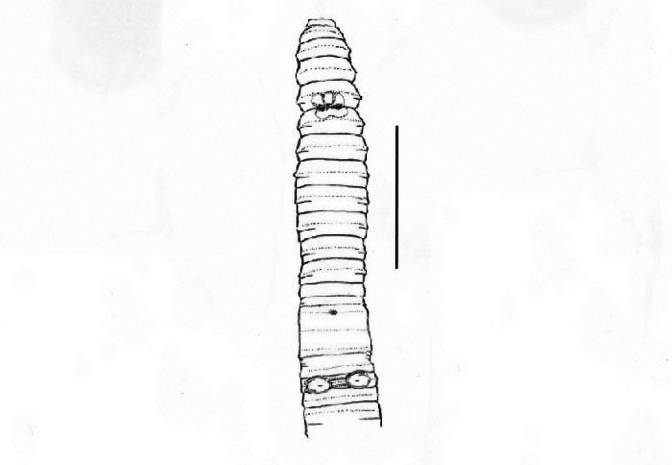
*Pheretima* sp. 1.: ventral view. Scale bar: 10 mm.

##### Description.

Medium purple-brown dorsal pigment much lighter on segmental equators. Dimensions 128 mm by 5 mm at segment X, 5.5 mm at XXX, 4.5 mm at clitellum, segment 122; body circular in cross-section. Setae 31 at VII, 52 at XX, 6 between male pores, dorsal setae larger, more widely spaced; setal formula AA:AB:YZ:ZZ = 2:2:4:5 at XIII. Clitellum annular XIV–XVI; setae invisible externally. First dorsal pore: 12/13. One pair of spermathecal pores in 5/6 at the 3^rd^ setal lines, 1 mm between spermathecal pores, 0.06 of the circumference apart, openings of copulatory bursa paired in XVIII at the 5^th^ to 6^th^ setal line, 2.4 mm between openings, 0.14 of the circumference apart. Genital markings lacking.

Septa 5/6–7/8 thick, 8/9 very thin, 9/10 absent, 10/11–11/12 thickened, 12/13 and 13/14 progressively thinner. Gizzard in VIII, intestine begins in XVI, small, paired lymph glands from XXX along the dorsal vessel, intestinal ceca simple originating in XXVII and extending anteriorly to XXIII, finger-shaped sac; typhlosole low-fold from XXVII, 30–32 intestinal blood vessels. Hearts in XI–XIII; X absent; IX lateral; VIII to gizzard; VII lateral with small connections to anterior edge of gizzard.

Ovaries and funnels in XIII, one pair of spermathecae in VI with nephridia on ducts, spermathecal ampulla elongate oval; barrel-shaped muscular duct shorter than ampulla; diverticulum chamber elongate oval, no iridescence, diverticulum stalk shorter than ampulla. Holandric male sexual system, testes, and funnels in ventrally paired sacs in X–XI. Seminal vesicles, two pairs in XI–XII with long apical dorsal lobe; small pseudo-vesicles in XIII; prostates in XVIII; stout muscular ducts, straight to top of copulatory bursae without stalked glands; copulatory bursae openings surrounded by three oval pads; wedge-shaped penis gradually increasing in height, laterally supported by bulbous projection from roof, pore at lateral tip of wedge.

##### Remarks.

This specimen probably represents another species from the secondary forests of central Catanduanes Island, indicating that our survey did not capture all the earthworm diversity in the area. This specimen had the following distinguishing combination of characteristics: hearts missing in X, septum 8/9 present, segmental equators lightly pigmented, head setae fewer than postclitellar setae (31 vs 52), and intestinal origin in XVI. We were not strict with respect to the setal count criterion because of the wide ranges in these counts for most species. Other species that match these characteristics are *P. batoensis* Hong & James, 2009, *P. bicolensis* Hong & James, 2009, *P. buhiensis* Hong & James, 2009, *P. gorasi* Hong & James, 2009, and *P. viracensis* Hong & James, 2009 (Table 2). All were from locations close to Mount Isarog or the same as the present taxon, in the case of *P. viracensis*. The reproductive pore spacing was much larger in *P. batoensis* and *P. viracensis*, and both lacked septa 8/9. Neither *P. buhiensis* nor *P. gorasi* had setae between the male openings (vs six), and the former had far more setae in the postclitellar segments. *Pheretima bicolensis* lacks typhlosoles and septa 8/9, both of which are present in *Pheretima* sp. 1.

## Supplementary Material

XML Treatment for Pheretima lapulapui

XML Treatment for Pheretima portiae

XML Treatment for Pheretima

## Additional information

### Use of AI

No use of AI was reported.

### Author ORCIDs

Yong Hong https://orcid.org/0000-0002-8093-9717

Samuel W. James https://orcid.org/0000-0001-6758-5726

### Data availability

All of the data that support the findings of this study are available in the main text.
